# Accumulation and removal of *Streptococcus mutans* biofilm on enamel and root surfaces *in vitro*

**DOI:** 10.2340/biid.v11.41059

**Published:** 2024-07-12

**Authors:** Anne Breivik, Aida Mulic, Amer Sehic, Håkon Rukke Valen, Simen Kopperud, Linda Stein, Qalbi Khan

**Affiliations:** a Inland Norway University of Applied Sciences, Elverum, Norway; b Nordic Institute of Dental Materials, Oslo, Norway; c Faculty of Dentistry, University of Oslo, Oslo, Norway; d Department of Clinical Dentistry, UiT The Arctic University of Norway, Tromsø, Norway

**Keywords:** Biofilm removal, Streptococcus mutans, enamel, root, root caries

## Abstract

**Objective:**

This study aimed to quantitatively investigate the accumulation of *Streptococcus mutans* biofilm on enamel and root surfaces and assess the amount of biofilm removal using (1) experimental toothpaste and (2) water, in a closed system of flow chamber.

**Methods:**

Eight sound premolars were embedded in epoxy resin and polished with silicon carbide grinding papers to display enamel and root surfaces. To mimic biofilm, cultures of *Streptococcus mutans* were prepared and grown on the tooth surfaces over night before they were exposed to either 2 liters of Milli Q water or 2 liters of 40% experimental toothpaste in the flow chamber. The amount of biofilm was measured and quantified in Fluorescence microscopy. Mean fluorescence values were recorded and analysed using Microsoft® Excel® (MS Excel 2016).

**Results:**

The ability to grow biofilm was equally present at both the enamel and root surfaces. The use of water and 40% experimental toothpaste showed a significant reduction of areas covered with biofilm on both enamel and root dentin in comparison to untreated surfaces (*p* < 0.01). Significantly more biofilm was removed from enamel compared to root surfaces when treated with either water and toothpaste (*p* < 0.01). Slightly less biofilm was removed by the use of water compared to toothpaste on both enamel and root dentin surfaces, although the differences were not statistically significant.

**Conclusion:**

The results indicate that less biofilm is removed from the root surfaces than enamel by the use of water and 40% experimental toothpaste in flow chamber. Assessing oral biofilm accumulation and monitoring biofilm formation on enamel and root dentin surfaces give oral health professionals important directions that could strenghten the significance of dental caries prevention. Improving older individuals’ oral hygiene practices should therefore be considered an important measure to prevent root caries.

## Introduction

Dental caries is a biofilm-mediated and multifactorial disease resulting in mineral loss of dental hard tissues. Carious lesions can be categorized according to their anatomical location on the tooth, on the coronal or root/cementum surfaces, respectively [1]. Although a range of bacteria are involved in the cariogenic flora in biofilm, *Streptococcus mutans* have been considered a key pathogen in the progression of dental caries [2–6]. Notwithstanding, root surfaces are at higher risk of biofilm-triggered mineral loss than enamel, as tooth substance of the root have lower mineral content than enamel [7]. As a result, the demineralization process occurs more rapidly on root surfaces than enamel [7,8]. Additionally, as the location of the root surface is close to the gingival margin and the cementoenamel junction, tooth cleaning in these areas is more difficult. Consequently, more biofilms are considered to be retained in these sites, making them more susceptible to dental caries [7,9,10].

Dental caries is the most widespread noncommunicable disease globally, affecting more than 2 billion people worldwide [11]. A systematic review on the global burden of untreated dental caries found that the oral disease is now peaking later in life, and has been shifting from childhood to adulthood [12]. The review stated that the prevalence and incidence of untreated dental caries increased after the age of 40, with a prevalence peak at 70 years of age due to the appearance of root caries [12]. Root caries is the dominant primary caries form in older people, particularly due to gingival recession caused by normal ageing process, but also as a result of periodontitis, which is highly prevalent among older individuals [13–19]. The exposed root surfaces are predisposed to oral microorganisms which cause demineralization of the cementum surfaces [20]. It is estimated that one-third of the geriatric population is affected by root caries [21], and as the proportion of elderly people is increasing worldwide, prevalence estimates will continue to increase in the future [8,22]. Efforts to prevent the burden of root caries in the elderly population are therefore strongly needed [8,23–25], particularly as the ability to remove biofilm often decreases as people age due to reduced manual dexterity, impaired vision or physical limitations associated with Parkinson’s disease or arthritis [9,26].

A common and effective method to reduce the amount of oral bacteria is mechanical removal with a toothbrush (manual or powered) supplied with fluoride toothpaste [23,27–29]. Interdental aids such as interdental brushes, dental floss or water flossers are recommended as supplements to remove biofilm from interproximal and subgingival areas [30]. Although the efficacy of various mechanical tools for removal of biofilm is well-documented [31–33], few studies have reported on how much biofilm removal is achievable through such mechanisms.

In addition, due to the irregular surface topography of the root surfaces, it has been argued that root surfaces have greater ability to retain and grow more oral biofilm compared to smooth enamel surfaces [10,34]. Dental enamel is an acellular, hard, avascular tissue, which consist of 96% inorganic material (hydroxyapatite nanocrystals), 3% water, and 1% organic component [35]. The enamel crystals form both prisms (rods) and interprisms (interrods) in the enamel, providing it its characteristic structure and strength. The rods and interrods cause the uneven microstructure of the surface enamel, which is especially noticeable when exposed to acid [36]. In contrast, the mineral content of dentin is approximately 50 vol% (hydroxyapatite minerals). The dentin is also a hydrated tissue, which is rich in both collagen and non-collagenous molecules (30 vol%). Consequently, dentin is considered structurally more intricate than enamel, involving dentinal tubules which contain a hypermineralized layer (peritubular dentin), and a softer intertubular dentin between them. Fluid from the pulp and cytoplasmic cell extensions of odontoblasts fill the dentinal tubes [36].

However, although adhesion of oral biofilm is assumed to differ between the root surfaces and enamel, the ability and grade of differences have not been widely demonstrated in controlled environments. Thus, assessing dental biofilm accumulation on both enamel and root surfaces and monitoring biofilm formation could give important directions that could strengthen the significance of caries prevention in older people. Therefore, the aim of this study was to quantitatively investigate the accumulation of *Streptococcus mutans* biofilm on enamel and root surfaces and assess the amount of biofilm removal using (1) experimental toothpaste and (2) water, in a closed system of flow chamber.

## Materials and methods

### Tooth surface preparations

**Figure 1:**
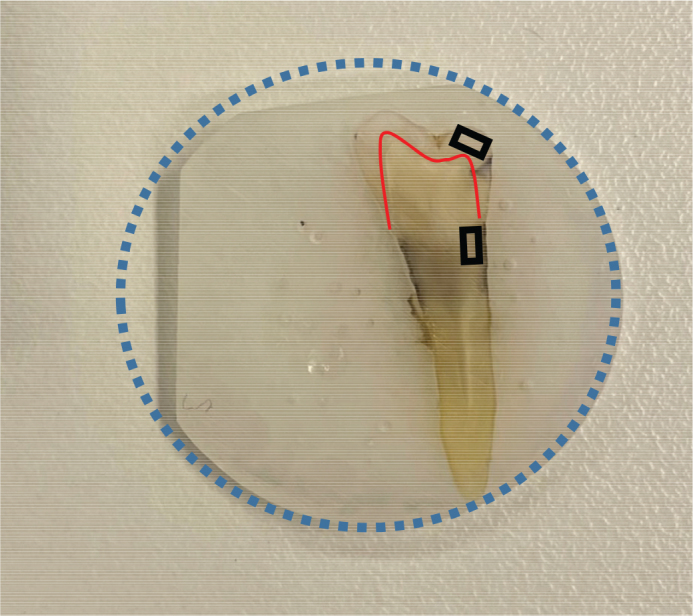
Premolars embedded in resin disks of 25 mm diameter (blue circle) were trimmed to fit in the flow chamber and to avoid movement. The red line depicts the enamel-dentin-junction. Images were approximately obtained from area of enamel (upper black rectangle) and root dentin (lower black rectangle).

Eight sound premolars from the Nordic Institute of Dental Materials (NIOM) toothbank (REK 2012/413) were placed in cylindrical shaped teflon molds (Ø 25 mm) and filled with epoxy resin (EpoFix; Struers, Rotherham, UK). After 24 hours, the molded teeth were polished with silicon carbide grinding papers (Struers, Ballerup, Denmark) to display enamel and root surface of either buccal or lingual aspect. The surfaces were polished with grinding paper with grain size 15 µm (Struers Waterproof SiC FEPA P# 1200, Ballerup, Denmark). The disk thickness was trimmed to ~2.5 mm (Figure 1). Prior to the experiments, the disks were rinsed in soap-water, and disinfected with 75% ethanol. They were then left in a 50 mL falcon tube containing a mixture of 75% ethanol diluted 1:3 in 10X phosphate buffered saline (Dulbecco’s PBS, DPBS, Lonza, Verviers, Belgium).

### Biofilm formation

Stock cultures of *Streptococcus mutans* (ATCC 700610) for experimental use were prepared from the batch stored at -70ºC freezer. An approximated amount of 10 µL bacteria, using a sterile plastic inoculation loop, was diluted in 10 mL of Brain Heart Infusion medium (BHI) (Oxoid Ltd, Basingstoke, UK) as a stock solution and incubated overnight (ON) (approximately 24 Hrs.) at 37ºC and 5% CO_2_ supplemented atmosphere.

The disks used for biofilm formation were washed with 75% ethanol for 2 minutes, using forceps, in circular movements. The disks were then air-dried before washing in Phosphate-buffered saline (PBS) (Lonza, Walkersville, MD, USA) for 2 minutes in same circular movements. The disks of teeth were then dried in separate wells before they were transferred into 6 wells of petri dishes (Corning Incorporated, ME, USA) containing a diluted (1:100) stock solution of *Streptococcus mutans* in BHI for ON in the laboratory cabinet at 37ºC and 5% CO_2_ supplemented atmosphere. Disks that were to serve as controls were placed in separate wells.

### Flow chamber

**Figure 2:**
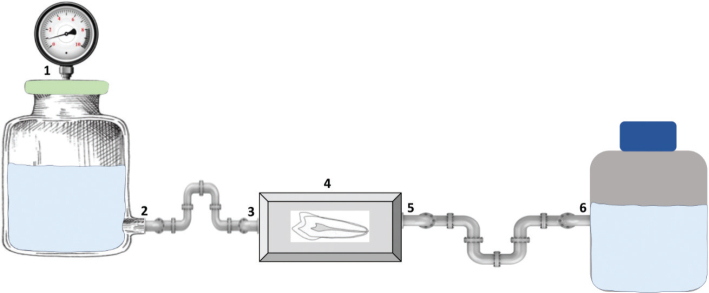
Flow chamber setup: (1) Air supply with pressure kept at approximately 1.5 bar – (2) Output valve with tank containing either water or 40% toothpaste – (3) Input valve – (4) Flow chamber containing tooth (embedded in resin disk) – (5) Outlet valve – (6) Waste container.

The disks were placed into the flow chamber (Figure 2) after the biofilm was formed overnight on the surface of interest. A parallel plate laminar flow chamber, milled from a single block of PMMA, was used for creating liquid flow directed towards and above the disks. The chamber was designed to fit a 50 mm × 25 mm glass slide, cut from ordinary microscope glass slides (VWR collection). A 5 mm thick quartz glass lid was sandwiched between the flow chamber and a rigid 8 mm aluminum plate. To fit the disks of teeth, the height of the chamber was fixed to 3 mm.

The biofilm covered disks were flushed with either 2 liter of Milli Q water or 40% experimental toothpaste in the flow chamber. Flow rate was kept at approximately 1 liter per minute, with a pressure at ~1.5 bar.

Four separate runs of all 8 teeth were conducted. The teeth were randomized in each experimental run. Two teeth were kept as positive control (‘Untreated’). Three teeth were subjected to Milli Q water treatment and three teeth were treated with 40% experimental toothpaste. Additionally, four tooth disks without biofilm formation, were served as negative control.

### Experimental toothpaste

Different toothpaste-dilutions were tested according to their ability to flow through the chamber-system without clogging the connected tubes and valves. The final flow rate and the pressure (~1.5 bar) were also a result of this consideration. A maximum amount of 40% toothpaste, diluted in Milli Q water, was found to be optimal for this. Hence a 40% experimental toothpaste was used in the trials described below. The toothpaste containing the main ingredients [37] (Table 1), was prepared, diluted, covered and then kept at room temperature on a magnetic stirrer before use.

**Table 1:** Weight amounts and the components used in the experimental toothpaste.

Composition	Weight
CaCO_3_ (Calcium carbonate)	80 g
SDS (sodium dodecyl sulphate)	3.9 g
CMC (Carboxymethyl cellulose)	2 g
Sorbitol	20 g
Glyserin/Glyserol	20 g
Distilled water	64 g

This produced 200 mL of toothpaste, which, prior to usage in this project, was diluted in 40% distilled water.

### Fluorescence microscopy

All eight teeth were measured and quantified for fluorescence biofilm on the root dentin and enamel. Before quantifying the biofilm formation in the fluorescence microscopy, all teeth were washed in PBS using forceps in circular movements. For bacterial viability, 1.5 µL of Filmtracer (LIVE/DEAD FilmTracer, Biofilm Viability Kit) was diluted in 1 mL 10X PBS. The samples were then incubated in room temperature, and covered with aluminimum foil for 15 minutes. The surfaces were then investigated using a fluorescence light microscope with excitation bandpass filter of 530–550 for red fluorescence of PI (Olympus BX51, Tokyo, Japan), where images using a 10x magnification were obtained, comparing the root dentin to the enamel for the same specimen.

### Fluorescence quantification

A Java-based image processing program, namely *ImageJ* (software version 1.51j, NIH, USA), was used to quantify fluorescence. The images were converted to 16-bit versions and adjusted for suitable level prior to measuring the mean value. Mostly, entire images were subjected to quantification. However, if the photographed images displayed cracks or artifacts, a standardized region of interest (ROI-tool) was utilised to eliminate this source of error. It this case, the comparing surface (enamel or root dentin) was matched for area of detection.

### Statistical analysis

Each run yielded two disks as positive control (untreated biofilm), three disks treated with water and another three treated with toothpaste. Two images (enamel and root) from the eight teeth specimens (16 images per run), through four different runs, provided a total number of 64 images, which were used in the statistical analysis.

Mean fluorescence values were recorded and analyzed using Microsoft® Excel® (MS Excel 2016). Average values and standard errors to denote a 95% confidence interval (CI) were calculated, and the effects of different treatments (water or toothpaste) on different surfaces (enamel or root dentin) were analyzed using one-tailed *t*-test with unequal variance. In this analysis, we set α < 0.01 to reduce the change of type I error and used Bonferroni correction to reduce the change of type II error.

## Results

### Fluorescence measurements of biofilm on tooth disks

**Figure 3:**
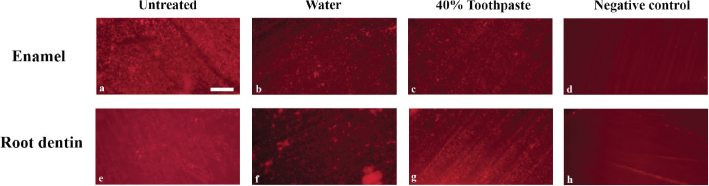
Layered *Streptococcus mutans* biofilm grown on a plane surface of enamel and root dentin (A, E). Reduced, but evenly distributed single layer of biofilm observed on plane enamel surfaces after water treatment. (B, F), with variating aggregation of biofilm. Little biofilm and less aggregations were observed on the surfaces after treatment with toothpaste (C,G). Negative controls, with no *Streptococcus mutans* biofilm, show neither biofilm or aggregations but lines after grinding (D, H). Scale bar of 200 µm is shown in image 3a.

Images from the fluorescence microscopy technique (with 10x magnification) appeared to display a multilayered formation of the *Streptococcus mutans* biofilm (Figure 3A, E). Treatment of disks with water flow showed reduced and evenly distributed single layered biofilm on the surface, including areas of accumulation (Figure 3B, F). Disks treated with 40% experimental toothpaste showed generally less amount of biofilm and areas with biofilm accumulation (Figure 3C, G). The negative control teeth without biofilm formation showed ground surfaces, but without biofilm formation (Figure 3D, H).

**Figure 4:**
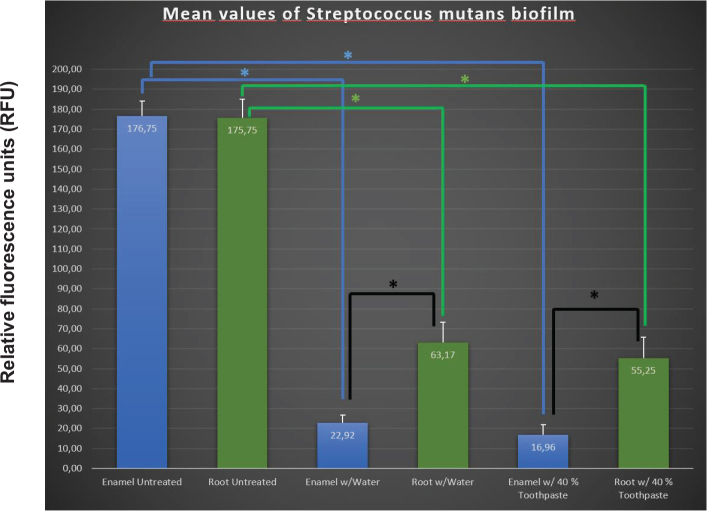
Mean fluorescence values (with errorbars denoting the 95% confidence interval determined by one-tailed *t*-test with unequal variance) of amount of *Streptococcus mutans* biofilm as measured in RFU, on plane root dentin and enamel surfaces. Treatment with water showed a reduction of biofilm area regarding both enamel and root dentin compared to untreated surfaces (*p-value < 0.01). Similar reduction is observed with 40% toothpaste for both enamel and root dentin (*p-value < 0.01). Difference between enamel and root dentin was found (*p-value < 0.01) when treating with both water and 40% toothpaste.

The quantified values of fluorescence biofilm are presented in Figure 4. Mean values with error bars denoting 95% confidence interval (CI) were determined by one-tailed *t*-test with unequal variance. *P*-values for compared groups are given in Table 2. The green bar graphs in Figure 4 represent mean values for biofilm on root dentin, and the blue bars show mean values of biofilm on enamel (Figure 4). The results showed that the ability to grow biofilm on enamel and root dentin was equally present at both tooth surfaces, and the differences were not found to be statistically significant (*p* > 0.45). Similar conclusion could be drawn regarding the use of water compared to 40% experimental toothpaste on enamel and root dentin, showing that although slightly less biofilm was removed using water compared to toothpaste on enamel (*p* > 0.18) and root dentin surfaces (*p* > 0.29), the results were not statistically significant. However, the results indicated that the use of water and toothpaste resulted in a significant reduction of area covered with biofilm on both enamel and root dentin, in comparison to untreated surfaces (*p* < 0.01). Correspondingly, significantly more biofilm was removed from enamel compared to root dentin surfaces using both water and toothpaste (*p* < 0.01).

**Table 2:** To determine significant difference in amount of biofilm between the compared groups, p-values (sig. diff < 0.01*) were calculated.

Compared mean values of biofilm	*p*-values	Corrected *p*-values
Untreated vs Water	9.66692E-10*	8.700228E-09
Untreated vs Toothpaste	1.05921E-10*	9.532890e-10
Water Vs Toothpaste	0.175943292	1.000000e+00
Untreated vs Water	1.22416E-07*	1.101744E-06
Untreated vs Toothpaste	5.73674E-08*	5.163066E-07
Water Vs Toothpaste	0.29883622	1.000000E+00
Untreated Enamel vs Untreated Root	0.467530711	1.000000E+00
Water Enamel vs Water Root	0.001261388*	1.135249E-02
Toothpaste Enamel vs Toothpaste Root	0.002494016*	2.244614E-02

## Discussion

This study aimed to quantitatively investigate the accumulation of *Streptococcus mutans* biofilm on enamel and root surfaces and assess the amount of biofilm removal using (1) experimental toothpaste and (2) water, in a closed system of flow chamber. The results demonstrated that the ability to grow biofilm was equally present at enamel and root surfaces, and there were no significant differences in the amount of biofilm accumulated on the two tooth surfaces (*p* = 0.45). These findings are not in congruence with research indicating that exposed root surfaces have binding properties different from enamel surfaces, which consequently could affect early biofilm formation on the adjacent enamel surface [10,34]. This discrepancy could be explained by our in vitro model, which is based on a simplified biofilm, without involving the complex oral environment of saliva, pellicle or any other microorganisms. However, our findings could still contribute to underscore the importance of biofilm removal from both enamel and root surfaces in clinical practice, especially as *Streptococcus mutans* particularly possesses the ability to colonize clean tooth surfaces at early stages [10]. More importantly, as research indicate that more biofilms are considered to be retained on root surfaces compared to enamel [34], and that the growth of microbiota on exposed root surfaces proceeds more rapidly than on enamel [10], plaque removal from root surfaces in clinical practice is of particularly great importance [23,38,39]. Additionally, as poor oral hygiene and the presence of plaque are considered important risk factors for root caries in older people [20,38], maintaining good oral hygiene practices and not allowing plaque to accumulate on exposed root surfaces are essential [38]. Hence, older individuals should be encouraged to brush their teeth twice a day [18,40], and for most elderly people, sulcular brushing with soft toothbrush (Bass method) is a preferable method for teeth cleaning [9]. Individuals with gingival recession should additionally be instructed to prevent further recession, which could involve the use of extra soft toothbrush, lighter brushing pressure or remodification of the brushing method [9]. Older people affected by diminished manual dexterity may additionally benefit from electric toothbrushes or manual customized toothbrushes [34,35]. For those being care-dependent, tooth brushing should be supported by caregivers [40,41], and dental professionals should provide necessary oral health care education [18,40].

The use of both water and 40% experimental toothpaste in flow chamber managed to remove biofilm from both enamel and root surfaces. A similar effect of water on plaque removal was found in a recent in vitro study, which tested the ability of water flossers to remove biofilm from training typodont teeth [33]. The study concluded that water flosser is an effective and appropriate oral hygiene device for cleaning teeth, preventing dental caries and maintaining oral hygiene [33]. Although our model does not have the same power as water floss devices, working in the pressure range of 50–90 psi. [29], our study has clinical relevance with regard to biofilms’ ability to adhere to surfaces of the teeth when treated with water or toothpaste under specific flow and pressure conditions. Notwithstanding, our model is not able to accurately simulate a traditional mechanical biofilm removal with toothbrushes or a professional biofilm removal with air/powder flow in the dental practice or even water flosser device. In future in vitro studies, we therefore suggest that parameters such as flow and pressure should be adjusted as much as possible to mimic the clinical aspect more closely, although factors such as valves and tubes naturally tend to pose limitations in laboratory studies.

Moreover, although not all biofilm was removed by either water or toothpaste, our results indicated that slightly more biofilm was removed on both tooth surfaces using experimental toothpaste compared with only water. This finding supports the evidence showing that particulate abrasive is a key ingredient for cleaning effectiveness in toothpaste [42]. Additionally, as the abrasivity of toothpaste largely depends on the amount of abrasive [43,44], it is reasonable to assume that a greater amount of plaque could have been removed from the tooth surfaces in this study, if the experimental toothpaste had not been diluted with water.

A main finding in this study was that significantly more biofilm was removed from enamel compared to root surfaces when treated with water and 40% experimental toothpaste. This indicates that the use of water and 40% experimental toothpaste in flow chamber is more able to efficiently remove biofilm from enamel in comparison to root surfaces. This could be explained by the irregular surface topography of the exposed root dentin surface, as the organization and structure of oral biofilm vary according to the sites where plaque forms [10]. Additionally, as poor oral hygiene and the presence of *Streptococcus mutans* are found to be associated with the formation of root caries [22,45], and because cleaning of root surfaces proves to be difficult due its location close to the gingival margin and cementoenamel junction [7,46], the importance of plaque removal from root surfaces is further reinforced. Thus, improving older individuals’ oral hygiene practices should therefore be considered an important measure to prevent root caries.

The single-species biofilm used in this study may be considered a limitation with regard to clinical comparison. The oral biofilm is both multi-species and interacts with the protein pellicle, probably adhering more firmly and hence not that easily removed as described in our model. As an attempt to mimic the biofilms attachment as in the oral environment, both coating with (sterile natural or artificial) saliva and introduction of 0.5% sucrose, prior to bacterial incubation, could have been implemented. These may have altered the attachment ability and hence the outcome of the study.

Furthermore, the cell concentration of the final bacterial suspension covering the disks was not measured. Optical density (OD) and bacterial count would have strengthened the model even further. However, this study focused on measurement of the remaining amount of biofilm covering the surface, not on the number of bacteria. Hence, the results remain informative in such scope.

Another aspect to take into consideration, is the number of samples used in this study. Our findings indicated that toothpaste treatment seemed to remove more biofilm than water, although the results were not supported by the statistical analysis as statistically significant. Hence, a larger sample could possibly have resolved this uncertainty.

## Conclusion

Based on the results of the present study, it can be concluded that the ability to grow biofilm was equally present at both enamel and root surfaces, as layered *Streptococcus mutans* biofilms grown on plane surfaces of enamel and root dentin were corresondingly equal.

It could further be concluded that significantly more biofilm was removed from enamel compared to root dentin surfaces by the use of both water and 40% experimental toothpaste in flow chamber. The results may give oral health professionals important directions that could strengthen the significance of dental caries prevention in the elderly population, and improvement of older individuals’ oral hygiene practices should be particularly considered in relation to root caries prevention.

## References

[1] Machiulskiene V, et al. Terminology of dental caries and dental caries management: consensus report of a workshop organized by ORCA and Cariology Research Group of IADR. Caries Res. 2019;54(1):7–14. 10.1159/000503309 31590168

[2] Oong EM, et al. The effect of dental sealants on bacteria levels in caries lesions: a review of the evidence. J Am Dent Assoc. 2008;139(3):271–8. 10.14219/jada.archive.2008.0156 18310731

[3] Ranganathan V, Akhila C. Streptococcus mutans: has it become prime perpetrator for oral manifestations? J Microbiol Exp. 2019;7:207–13. 10.15406/jmen.2019.07.00261

[4] Matsumoto-Nakano M. Role of Streptococcus mutans surface proteins for biofilm formation. Jap Dent Sci Rev. 2018;54(1):22–9. 10.1016/j.jdsr.2017.08.002 29628998 PMC5884221

[5] Fure S. Ten-year cross-sectional and incidence study of coronal and root caries and some related factors in elderly Swedish individuals. Gerodontology. 2004;21(3):130–40. 10.1111/j.1741-2358.2004.00025.x 15369015

[6] Sánchez-García S, et al. A prediction model for root caries in an elderly population. Community Dent Oral Epidemiol. 2011;39(1): 44–52. 10.1111/j.1600-0528.2010.00569.x 20735446

[7] Balhaddad AA, et al. Pronounced effect of antibacterial bioactive dental composite on microcosm biofilms derived from patients with root carious lesions. Front Mater. 2020;7. 10.3389/fmats.2020.583861

[8] Pentapati KC, Siddiq H, Yeturu SK. Global and regional estimates of the prevalence of root caries – systematic review and meta-analysis. Saudi Dent J. 2019;31(1):3–15. 10.1016/j.sdentj.2018.11.008 30705564 PMC6349959

[9] Razak PA, et al. Geriatric oral health: a review article [Internet]. J Int Oral Health. 2014 [cited 07-02-2023];6(6):110–16. Available from: https://www.ncbi.nlm.nih.gov/pmc/articles/PMC4295446/25628498 PMC4295446

[10] Yu OY, et al. Dental biofilm and laboratory microbial culture models for cariology research. Dent J (Basel). 2017;5(2):21. 10.3390/dj5020021 29563427 PMC5806974

[11] World Health Organization. Global oral health status report. Towards universal health coverage for oral health by 2030 [Internet]. 2022. Available from: https://www.who.int/publications/i/item/9789240061484 [cited 22 Jan 2024]

[12] Kassebaum NJ, et al. Global burden of untreated caries: a systematic review and metaregression. J Dent Res. 2015;94(5):650–8. 10.1177/0022034515573272 25740856

[13] Zhang N, et al. Development of a multifunctional adhesive system for prevention of root caries and secondary caries. Dent Mater. 2015;31(9):1119–31. 10.1016/j.dental.2015.06.010 26187532 PMC4665983

[14] Heasman PA, et al. Gingival recession and root caries in the ageing population: a critical evaluation of treatments. J Clin Periodontol. 2017;44(S18):S178–93. 10.1111/jcpe.12676 28266119

[15] Hayes M, et al. Risk indicators associated with root caries in independently living older adults. J Dent. 2016;51:8–14. 10.1016/j.jdent.2016.05.006 27208875

[16] Cai J, et al. Status and progress of treatment methods for root caries in the last decade: a literature review. Aust Dent J. 2018;63(1):34–54. 10.1111/adj.12550 28833210

[17] Qutieshat A, et al. Perspective and practice of root caries management: a multicountry study – part I. J Conserv Dent. 2021;24(2):141–7. 10.4103/jcd.jcd_19_21 34759579 PMC8562823

[18] Paris S, et al. How to intervene in the caries process in older adults: a joint ORCA and EFCD expert Delphi consensus statement. Caries Res. 2020;54(5–6):459–65. 10.1159/000510843 33291110

[19] Takahashi N, Nyvad B. Ecological hypothesis of dentin and root caries. Caries Res. 2016;50(4):422–31. 10.1159/000447309 27458979

[20] Sen S, et al. Prevalence and risk factors of root caries in the geriatric population in the rural sector. J Fam Med Prim Care. 2020;9(2):771–6. 10.4103/jfmpc.jfmpc_1053_19 PMC7114028 32318418

[21] Griffin SO, et al. Estimating rates of new root caries in older adults. J Dent Res. 2004;83(8):634–8. 10.1177/154405910408300810 15271973

[22] Zhang J, et al. Risk predictors of dental root caries: a systematic review. J Dent. 2019;89:103166. 10.1016/j.jdent.2019.07.004 31301318

[23] Gati D, Vieira AR. Elderly at greater risk for root caries: a look at the multifactorial risks with emphasis on genetics susceptibility. Int J Dentistry. 2011;2011:e647168. 10.1155/2011/647168 PMC3133477 21754932

[24] Sundaragopal N, Hou L, Enciso R. Efficacy of non-invasive and minimally invasive techniques for the prevention/management of root caries in older adults – a literature review. Dent Rev. 2022;2(4):100061. 10.1016/j.dentre.2022.100061

[25] Tonetti MS, et al. Dental caries and periodontal diseases in the ageing population: call to action to protect and enhance oral health and well-being as an essential component of healthy ageing – consensus report of group 4 of the joint EFP/ORCA workshop on the boundaries between caries and periodontal diseases. J Clin Periodontol. 2017;44(S18):S135–44. 10.1111/jcpe.12681 28266112

[26] Shin NR, Choi JS. Manual dexterity and dental biofilm accumulation in independent older adults without hand disabilities: a cross-sectional study. Photodiagnosis Photodyn Ther. 2019;25:74–83. 10.1016/j.pdpdt.2018.11.007 30439530

[27] Figuero E, et al. Mechanical and chemical plaque control in the simultaneous management of gingivitis and caries: a systematic review. J Clin Periodontol. 2017;44(S18):S116–34. 10.1111/jcpe.12674 28266113

[28] Vyas T, et al. Chemical plaque control – a brief review. J Fam Med Prim Care. 2021;10(4):1562–8. 10.4103/jfmpc.jfmpc_2216_20 PMC8144784 34123892

[29] Abdellatif H, et al. Comparison between water flosser and regular floss in the efficacy of plaque removal in patients after single use. Saudi Dent J. 2021;33(5):256–9. 10.1016/j.sdentj.2021.03.005 34194188 PMC8236551

[30] Mancinelli-Lyle D, et al. Efficacy of water flossing on clinical parameters of inflammation and plaque: a 4-week randomized controlled trial. Int J Dent Hyg. 2023;21(4):659–68. 10.1111/idh.12770 37753575

[31] Elkerbout TA, et al. How effective is a powered toothbrush as compared to a manual toothbrush? A systematic review and meta-analysis of single brushing exercises. Int J Dent Hyg. 2020;18(1):17–26. 10.1111/idh.12401 31050195 PMC7004084

[32] Thomassen TMJA, Van der Weijden FGA, Slot DE. The efficacy of powered toothbrushes: a systematic review and network meta-analysis. Int J Dent Hyg. 2022;20(1):3–17. 10.1111/idh.12563 34877772 PMC9303421

[33] Wang Y, et al. Efficient removal of dental plaque biofilm from training typodont teeth via water flosser. Bioengineering (Basel). 2023;10(9):1061. 10.3390/bioengineering10091061 37760162 PMC10525826

[34] Rüdiger Stefan G, Dahlén G, Carlen A. Protein and bacteria binding to exposed root surfaces and the adjacent enamel surfaces in vivo. Swed Dent J. 2015;39(1):11–22. 26529838

[35] Malcangi G, et al. Analysis of dental enamel remineralization: a systematic review of technique comparisons. Bioengineering (Basel). 2023;10(4):472. 10.3390/bioengineering10040472 37106659 PMC10135549

[36] Shahmoradi M, et al. Fundamental structure and properties of enamel, dentin and cementum. Adv Calc Phosphate Biomater. 2014;2:511–47. 10.1007/978-3-642-53980-0_17

[37] Podobii O, Ladonko M. Optimization of the recipe of toothpaste by carrageenan addition. UJFS. 2017;5(1): 63-70. 10.24263/2310-1008-2017-5-1-9

[38] Zhang J, Lo ECM. Epidemiology of dental root caries: a review of risk factors. Front Oral Maxillofac Med. 2020;2. 10.21037/fomm.2020.03.02

[39] Rodrigues J, et al. Prevention of crown and root caries in adults. Periodontology 2000. 2011;55:231–49. 10.1111/j.1600-0757.2010.00381.x 21134238

[40] Charadram N, et al. Development of a European consensus from dentists, dental hygienists and physicians on a standard for oral health care in care-dependent older people: an e-Delphi study. Gerodontology. 2021;38(1):41–56. 10.1111/ger.12501 33073408

[41] Mac Giolla Phadraig C, et al. The complexity of tooth brushing among older adults with intellectual disabilities: findings from a nationally representative survey. Disabil Health J. 2020;13(4):100935. 10.1016/j.dhjo.2020.100935 32439304

[42] Sarembe S, et al. Influence of the amount of toothpaste on cleaning efficacy: an in vitro study. Eur J Dent. 2023;17(2):497. 10.1055/s-0042-1747953 35785824 PMC10329550

[43] Johannsen G, et al. The importance of measuring toothpaste abrasivity in both a quantitative and qualitative way. Acta Odontol Scand. 2013;71(3–4):508–17. 10.3109/00016357.2012.696693 22746180 PMC3665314

[44] Wang C, et al. Particle size effects on abrasion, surface polishing and stain removal efficacy in a tooth model system. Biotribology. 2021;28:100196. 10.1016/j.biotri.2021.100196

[45] Zhang J, et al. Factors associated with dental root caries: a systematic review. JDR Clin Trans Res. 2020;5(1):13–29. 10.1177/2380084419849045 31145661

[46] Närhi T, Syrjälä AM. Dental diseases and their treatment in the older population. Tidende. 2017;127(1): 42-48. 10.56373/2017-1-7

